# Remodelling After Percutaneous Pinning for Slipped Capital Femoral Epiphysis: The Influence of Transphyseal Screw Position

**DOI:** 10.3390/children13030404

**Published:** 2026-03-14

**Authors:** Joeri Slobbe, Cornelis L. P. van de Ree, Johannes H. J. M. Bessems, Jaap J. Tolk

**Affiliations:** Department of Orthopaedics and Sports medicine, Erasmus MC-Sophia Children’s Hospital, 3015 GD Rotterdam, The Netherlands

**Keywords:** slipped capital femoral epiphysis, SCFE, percutaneous fixation, remodelling

## Abstract

**Highlights:**

Screw position was found to have an influence on the process of remodelling in patients with SCFE. This could potentially lead to better outcomes for patients with SCFE; however, the effect observed was relatively small, and further research is warranted. Caution should be taken to avoid compromising physeal fixation or penetrating the joint when adjusting SCFE screw placement.

**What are the main findings?**

• The amount of remodelling after in situ fixation for SCFE is influenced by the severity of initial slip and a more lateral transphyseal screw position.

**What are the implications of the main findings?**

• Optimisation of screw position has the potential to guide growth after SCFE and reduce residual deformity at skeletal maturity. However, the guided growth effect observed in this study is too small to justify a change in current clinical practice.

**Abstract:**

**Introduction:** Slipped capital femoral epiphysis (SCFE) is commonly treated using percutaneous in situ fixation. After screw fixation, remodelling of the proximal femur can occur; however, the factors influencing this process are poorly understood. This study aimed to measure the amount of remodelling after in situ SCFE fixation and determine the influence of the transphyseal screw position across the physis. **Methods:** In this retrospective study, all eligible patients with SCFE who had percutaneous screw fixation at Erasmus MC—Sophia Children’s Hospital between 2012 and 2020 were included. The amount of remodelling was determined by measuring the Southwick angle, alpha angle and displacement from Klein’s line directly after screw fixation and at final follow-up. Transphyseal screw position was measured through AP and frog-leg lateral radiographs by measuring the placement of the centre of the screw in relation to the centre of the epiphysis. A linear mixed model was used to determine factors influencing the amount of remodelling. **Results:** 86 patients with 96 affected hips were included; the mean age was 12.4 (±2.0) years at surgery, and the mean follow-up duration was 3.7 (±2.0) years. All measurements showed significant remodelling at follow-up compared to baseline. Over the follow-up period, the mean change in Southwick angle was 4.6° (95% CI: 2.5; 6.7, *p* < 0.001), the mean change in Alpha angle was 10.4° (95% CI: 7.3; 13.5, *p* < 0.001) and the mean change in displacement from Klein’s line was −1.2 mm (95% CI: −1.7; −0.61, *p* < 0.001). Linear mixed model analyses showed that remodelling was significantly correlated with deformity at baseline for all measurements. Also, a more lateral screw position was significantly correlated with more improvement in displacement from Klein’s line (estimate: −4.2, 95% CI: −8.0 to −0.5). However, the effect observed was relatively small. **Conclusions:** A statistically significant amount of remodelling was measured after percutaneous screw fixation for patients with SCFE. The amount of remodelling was relatively limited, but was shown to be influenced by the severity of the initial slip and a more lateral transphyseal screw position.

## 1. Introduction

Slipped capital femoral epiphysis (SCFE) is a disorder of the proximal femur that affects adolescent children. In patients with SCFE, the proximal neck and shaft of the femur move anteriorly and in external rotation in relation to the proximal femoral epiphysis [1,2]. SCFE is also the most common hip disorder in adolescents [3]. An annual incidence of 11.6 per 100,000 children in the Dutch population has been reported [4]. Obesity is a major risk factor for SCFE [5]. In line with the increasing rate of childhood obesity, the incidence of SCFE is also rising [6].

The most commonly performed primary treatment for SCFE is a percutaneous screw fixation to prevent progressive deformity of the proximal femur [7]. It has been shown that a fully threaded screw of 6.5 mm or greater placed at least four threads across the physis is sufficient to prevent further progression of the slip [8]. However, an alteration of the proximal femur’s morphology usually remains, potentially resulting in femoroacetabular impingement (FAI) and early hip osteoarthritis [9]. Because of the residual proximal femoral deformity after percutaneous fixation, capital realignment procedures are advocated for by some, aiming to lower the future risk of FAI [10]. Nevertheless, with this procedure, concerns regarding higher rates of significant postoperative complications exist [11].

Previous studies have found that after the treatment of SCFE with percutaneous screw fixation, the proximal femur can correct some of the abnormal morphology, a process called remodelling [12,13]. It has been described that details of screw type and placement influence residual growth and remodelling of the proximal femur in patients with SCFE [14,15]. For other indications, such as hip migration in cerebral palsy and developmental dysplasia of the hip, eccentric proximal femoral screw placement has been shown to be effective in correcting deformity, a phenomenon also known as guided growth [16]. For these indications, the screw is usually placed on the medial side of the physis, which results in progressive varus of the proximal femur. In patients with SCFE, the usual femoral shaft displacement is in varus and procurvatum. Hypothetically, a screw placed in the anterolateral quadrant of the epiphysis could have a growth modulatory effect that would gradually reduce the SCFE deformity. To the best of our knowledge, no detailed analysis on the influence of screw positioning on remodelling after SCFE has been reported to date.

We aimed to determine the amount of remodelling of the proximal femur after percutaneous fixation with a transphyseal screw. Furthermore, the study aimed to identify predictors for the amount of remodelling, including the transphyseal screw position. We hypothesised that a more anterior and laterally placed screw would result in more remodelling of the proximal femur.

## 2. Materials and Methods

This study was a monocentric retrospective study evaluating clinical and radiologic data of patients with SCFE. A cohort of consecutive patients treated with percutaneous screw fixation for both stable and unstable SCFE in the period of 2012–2020 in one academic hospital with at least 1 year of radiological follow-up was included. All patients included were treated with in situ screw fixation, regardless of the degree of slip. Patients were excluded when they suffered from an underlying metabolic or skeletal disease, when the radiographic follow-up was less than one year, or when the quality of the radiographs was insufficient for accurate measurements. Patients were also excluded if they had ipsilateral surgical treatment changing the proximal femur anatomy, such as an osteotomy of the proximal femur or cam resection, within the follow-up period. In patients with bilateral SCFE, both hips were included separately.

For the included patients, clinical data regarding age at surgery, sex, height, weight, side of SCFE, contralateral SCFE, other operations and complications were extracted. Clinical and radiographic complications during the follow-up period were recorded, including the occurrence of avascular necrosis of the femoral head, chondrolysis, osteoarthritis and screw malposition.

**Figure 1:**
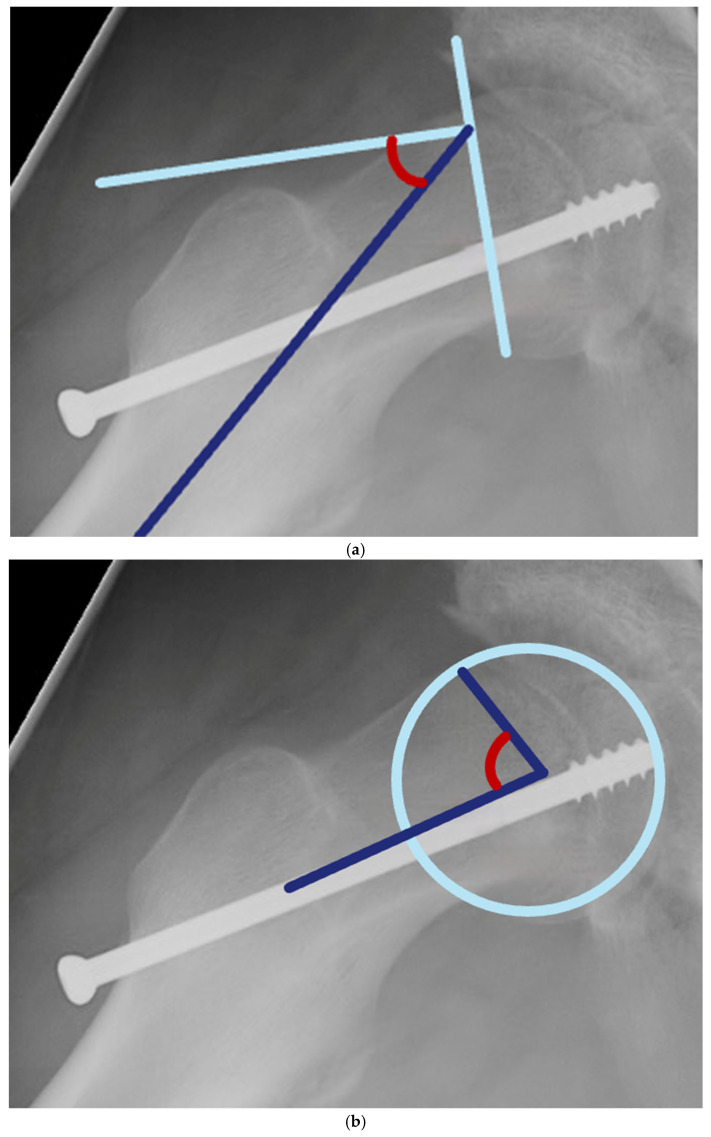

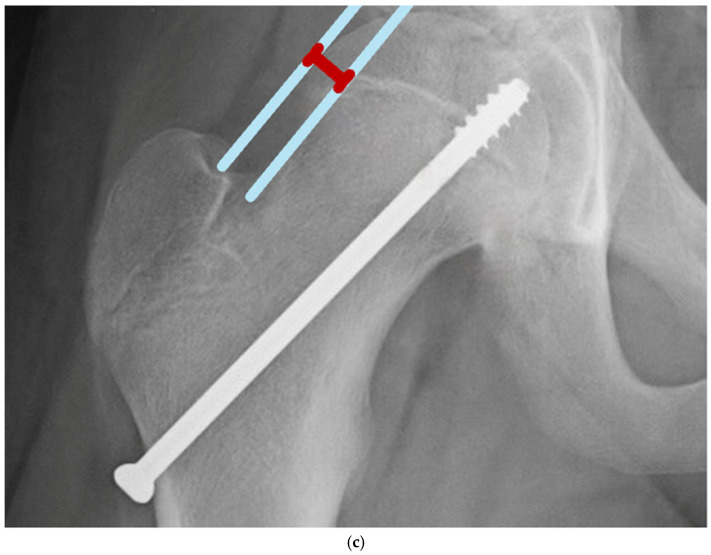
(**a**) The Southwick angle, a common measurement to determine the severity of a slip, was measured on the frog-leg lateral radiographs as the angle between a line drawn perpendicular to the line between the anterior and posterior tips of the physis, and a line along the axis of the femur. (**b**) The alpha angle was defined as the angle between two lines drawn from the centre of the femoral head, with one line being drawn longitudinal to the femoral neck and the other line being drawn to the point where the femoral head or neck breaks the circle of the femoral head anteriorly on the frog-leg lateral radiograph. (**c**) Displacement from Klein’s line was measured as the distance between two parallel lines drawn on the AP radiograph. The first was along the superior border of the femoral neck, and the second along the superolateral margin of the epiphysis.

To determine the amount of deformity at baseline and the amount of remodelling of the proximal femur, measurements were performed on the first postoperative radiographs and at the final follow-up. Measurements were performed using the Philips Vue PACS system version 12.2.8.300.0185 (2023). On the frog-leg lateral radiographs, the Southwick angle and alpha angle were measured. The hospital where the study was performed uses a standardised method for radiological measurements to prevent differences in pelvic tilt and leg rotation. In this study, frog-leg lateral radiographs were performed bilaterally, where the X-rays are centred at the pubic symphysis. On the anterior–posterior (AP) radiographs, measurements of the displacement from Klein’s line were made. The Southwick angle, a common measurement to determine the severity of a slip, was measured according to its original description. (Figure 1a) [17]. The alpha angle, used for the radiological evaluation of signs of FAI, was measured by the method of Nötzli et al. (Figure 1b) [18]. Both the Southwick angle and alpha angle’s intra- and interobserver reliability have been described as good [19,20]. Displacement from Klein’s line was measured in millimetres. (Figure 1c) [21]. This measurement has substantial intraobserver reliability and moderate interobserver reliability [22].

**Figure 2:**
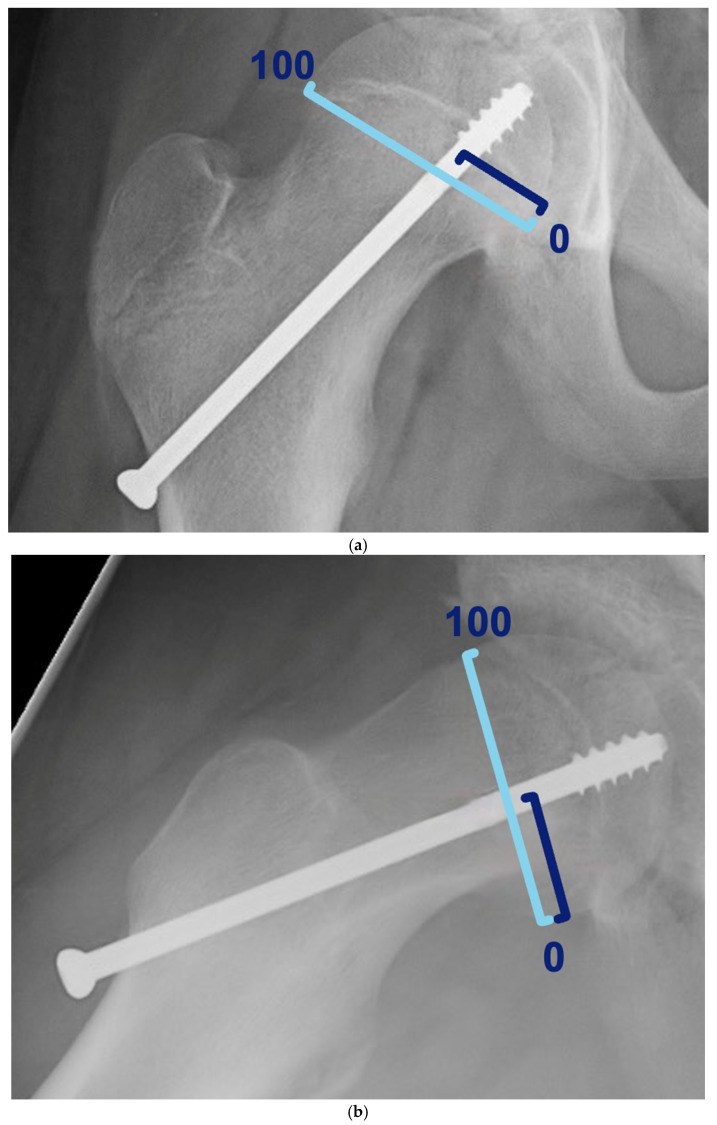
(**a**) The position of the screw on the AP radiograph was determined by drawing a line from the lateral to the medial edge of the physis, and a second line from the middle of the screw to the medial part of the physis. (**b**) The position of the screw on the frog-leg lateral radiograph was determined similarly, using the anterior and posterior edges of the physis and a line from the middle of the screw to the posterior edge of the physis.

The transphyseal screw position was measured as originally described to measure the screw position for hips in cerebral palsy patients in both the AP and frog-leg lateral radiographs (Figure 2a,b). This method has excellent intra- and interobserver reliability [23]. Following this method, the epiphyseal part of the physis was measured to determine the screw position. Using these measurements, the percentage of the physis medially to the screw was calculated, ranging from 0 (medial border of the physis) to 100 (lateral border). Similarly, a ratio was determined by dividing the part posterior to the screw by the total distance of the physis on the frog-leg lateral radiographs, ranging from 0 (posterior border of the physis) to 100 (anterior border). Based on these two measurements, the distance from the screw to the centre of the physis and the location where the screw penetrates the physis were determined.

Data were analysed using IBM SPSS Statistics software, version 28.0.1.0. All baseline data were normally distributed [24]. The changes in measurements between directly after screw fixation and the last follow-up radiographs were compared using Student’s *t*-test. We performed linear mixed model analyses to identify factors influencing the amount of remodelling. We corrected for the intra-individual correlation of outcome in patients with bilateral hip involvement by analysing the patient as a subject variable within the model. The patient was used as a random effect within the model to allow for a random intercept per patient. The covariance structure assumed in this model was unstructured. The radiological measurements were used as the dependent variable. The models were checked for linearity by creating scatterplots comparing predicted values to residuals, and for normality by analysing Q-Q plots. Linear mixed models were created for each of the 3 radiological measurements separately (Southwick angle, displacement from Klein’s line and alpha angle). For each analysis, gender, age at surgery, follow-up duration, screw position and the baseline measurement of the respective radiological measurement were used as predictor variables. Confidence intervals were set at 95%, and a *p*-value ≤ 0.05 was considered statistically significant.

## 3. Results

A total of 96 hips from 86 patients who had screw fixation for SCFE from 2012 until 2020 were eligible for inclusion. The patient characteristics and radiological outcomes at baseline are shown in Table 1. The average follow-up duration of the patients was 3.7 ± 2.0 (3.3; 4.1) years.

**Figure 3:**
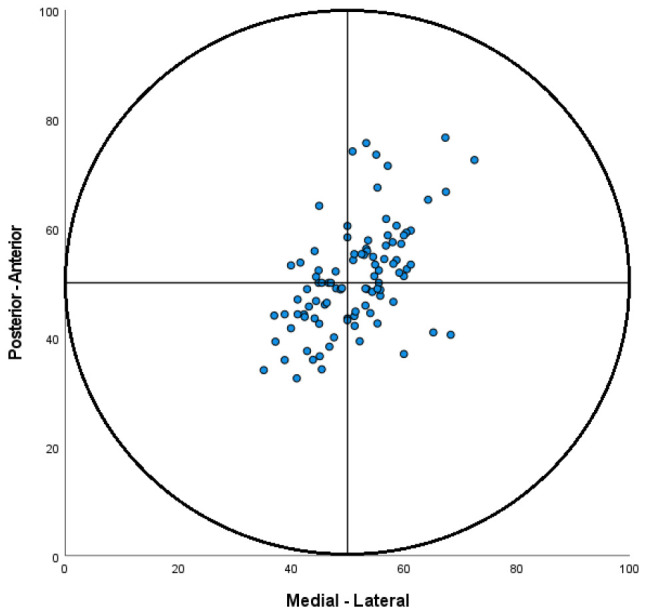
Visualisation of distribution of the position of the screws in the physis. The X-axis represents the distribution on the AP radiograph on a scale of 0 to 100, and the Y-axis the distribution on the frog-leg lateral radiograph. Each dot represents an individual patient’s transphyseal screw position. The circle represents the physis.

The distribution of the screw positioning in the physis at baseline is visualised in Figure 3. For all radiographic measurements, a significant decrease in deformity between baseline and follow-up was observed, reflecting the amount of remodelling (Table 2).

The amount of Southwick angle remodelling showed to be significantly influenced by duration of follow-up and the Southwick angle at baseline (Table 3).

The amount of remodelling in alpha angle was significantly influenced by follow-up duration and baseline alpha angle (Table 4). The amount of remodelling in displacement from Klein’s line was significantly influenced by follow-up duration, baseline displacement from Klein’s line, and the position of the screw on AP, where more laterally placed screws are associated with greater displacement from Klein’s line at follow-up (Table 5). Within the distribution of screw placement in this cohort, the influence of positioning a screw 1% more laterally leads to 0.042 mm more expected remodelling of the displacement from Klein’s line.

Three (3.1%) of the hips showed osteoarthritis at follow-up, and four (4.2%) hips developed avascular necrosis. One patient who developed avascular necrosis had a relatively posterior screw position (0.34 on the frog-leg lateral view). However, all other patients that developed avascular necrosis or osteoarthritis had screws placed relatively close to the centre on both AP and frog-leg lateral (all between 0.45 and 0.55 on both AP and frog-leg lateral views). Of the patients that developed avascular necrosis, three had unstable SCFE at presentation, while one was stable, two had a moderate slip (Southwick angle of 30–50°), and two had a severe slip (Southwick angle > 50°). At the moment of reviewing the data, eight patients had undergone a proximal femur osteotomy. For all patients, the indication for the osteotomy was persistent pain and functional limitation related to anatomical deformity after in situ screw fixation. These eight patients were excluded from the study. In these eight patients, the average Southwick angle at baseline was 52°, the average alpha angle at baseline was 80°, and the average displacement from Klein’s line at baseline was 3.0 mm. The average screw placement in this group was 0.55 on the AP view and 0.53 on the frog-leg lateral view. Other baseline characteristics were similar to those of the included patients. In 15 (15.6%) cases, the screw remained symptomatic, and removal was indicated after physeal closure. Further complications included a broken k-wire (n = 1), perforation of the caput femoris by the screw (n = 1) and screw loosening during follow-up (n = 1). During the follow-up period, there were no patients with chondrolysis. There were no patients in this cohort who underwent total hip arthroplasty during the follow-up period.

## 4. Discussion

This is the first study that has analysed the correlation between the transphyseal screw position and the amount of remodelling after percutaneous fixation for SCFE treatment. We found that a significant amount of remodelling of the proximal femur is expected after treatment of SCFE with percutaneous fixation. The amount of remodelling is correlated with the severity of initial displacement deformity and partially by transphyseal screw position.

Previously, it has been debated whether growth continues after percutaneous fixation for SCFE. In smaller series, both complete physeal closure and continued proximal femur growth have been reported [25,26,27]. Other studies suggest that factors beyond longitudinal growth contribute to the extent of angular remodelling [28]. The improvement in proximal femoral deformity observed in this study supports that growth persists, at least to some extent, following in situ fixation. This continued growth is likely to contribute to SCFE remodelling.

The amount of remodelling in this study was slightly greater than reported in previous reports in which similar conventional transphyseal screws were used [14]. This difference might be explained by the longer follow-up period in the present study. It must be noted that overall, remodelling was relatively limited. It has been shown that a more severe slip leads to a higher incidence of FAI [9]. Overall, the remaining deformity at follow-up is still considered a moderate slip. However, some patients did improve from a moderate to a mild slip after remodelling, potentially reducing their risk of FAI [29].

So far, the optimal screw position for percutaneous fixation is considered to be in the centre of the physis [30]. Our finding that screw position influences remodelling might challenge this dogma. However, we did observe this effect only for remodelling measured in displacement from Klein’s line. Furthermore, the absolute influence of screw position change was relatively small; for each 1% more lateral position, 0.042 more remodelling of displacement from Klein’s line can be expected. In the spread of the screw placements on AP in this study (0.37–0.68), this would give a theoretical increase in the remodelling effect on the displacement of Klein’s line of the most lateral screw by 1.3 mm when compared to the most medial screw, and an increase of 0.8 mm when compared to screws placed in the centre. While this finding is statistically significant, it is not proven, and it is not very likely that this has clinical significance. The small effect size observed might be explained by the relatively limited variation in transphyseal screw position in our cohort. Almost no screws were positioned in the hypothetically ‘optimal’ anterolateral quadrant (Figure 3). It also must be noted that for the Southwick angle and alpha angle, no relationship between screw position and remodelling was found. Both these measurements were performed on frog-leg lateral radiographs. Positioning variability in the frog-leg lateral plane is known to affect radiographic measurements [31]; this may have obscured potential guided growth effects.

In most patients, the screw was positioned relatively near the centre of the physis, and the variability of the screw placements was limited. (Figure 2) This may have limited the guided growth effect that could be observed in this study. Hypothetically, screws positioned more eccentrically anterior and lateral than in this cohort would induce a greater growth modulation effect. However, eccentric transphyseal screw position may not be without risks. Since the theoretical ‘optimal’ placement would be anterolateral of the physeal centre, it could be more difficult to fluoroscopically visualise during surgery. This could increase the chance of intra-articular screw penetration and should be taken into account when adapting the screw position. Obviously, it remains unchallenged that strong physeal fixation is the primary goal. A large-diameter screw and at least four threads across the physis have been recommended to prevent progressive slippage after fixation [8,32,33].

This study had some limitations. Due to the study design, the correlation between radiological findings and patient-experienced outcomes could not be analysed. As radiological parameters do not always fully align with symptoms and patient-reported outcomes, it is essential to include this perspective in future studies [34]. Slip stability was unfortunately not reported consistently in most cases and therefore could not be analysed in this study. Also, while this study used a standardised method of performing radiographs, positioning errors cannot be ruled out. Frog-leg lateral radiographs are especially susceptible to positioning variability, leading to measurement errors when compared to 3D measurements [31]. Future studies with 3D imaging are warranted to ensure accurate measurements and verify the potential guided growth effect of transphyseal SCFE screws. Furthermore, a prolonged follow-up period would be needed to elucidate the effects of remodelling on long-term clinical outcomes like FAI and osteoarthritis. While it is known that smaller slip angles at baseline decrease the chance of needing a total hip arthroplasty later, it is uncertain whether higher remodelling will have a similar effect [35]. Finally, eight patients who underwent a proximal femur osteotomy were excluded. The average radiological measurements at baseline showed more severe SCFE deformity in this group compared to the included patients. It is sensible that secondary surgery was performed in more severe cases, but this may have introduced selection bias in our cohort.

## 5. Conclusions

In conclusion, remodelling of the proximal femur can be expected after percutaneous fixation for the treatment of SCFE. The amount of remodelling is significantly predicted by the severity of the deformity. In this study, more laterally placed screws on the AP radiograph were related to more remodelling during growth, but the magnitude of this effect was very limited. Because in this study, all screws were placed relatively close to the centre of the physis, more research would be needed to determine if even more eccentrically placed screws would lead to more remodelling. The study findings do not yet support a change in screw position in routine clinical practice.

## Figures and Tables

**Table 1:** Baseline characteristics (*n* = 86 patients/96 hips).

Age at surgery (years)	12.4 ± 2.0 (12.0; 12.8)
Follow-up (years)	3.7 ± 2.0 (3.3; 4.1)
Body mass index (kg/m^2^)	24.2 ± 4.3 (23.2; 25.2)
Side (left)	56 (58.3%)
Bilateral	23 (26.7%)
Prophylactic contralateral screw fixation	9 (9.4%)
Gender (male)	45 (46.9%)
Screw position on AP (0 = medial, 1 = lateral)	0.51 ± 0.08 (0.50; 0.53)
Screw position on frog-leg lateral (0 = posterior, 1 = anterior)	0.51 ± 0.10 (0.49; 0.53)
Screw distance from centre of the physis (mm)	4.9 ± 3.0 (4.3; 5.5)

Values are presented as mean ± standard deviation (range) or number (% of *n* = 96).

**Table 2:** Difference between radiological measurements at baseline and follow-up.

	Mean		Mean Difference	*p*-Value
	Baseline	Follow-Up		
Southwick angle (°)	36.5 ± 12.3	31.9 ± 10.9	4.6 (2.5; 6.7)	<0.001
Alpha angle (°)	72.6 ± 15.1	62.1 ± 16.5	10.4 (7.3; 13.5)	<0.001
Displacement from Klein’s line (mm)	4.1 ± 2.7	5.3 ± 3.2	−1.2 (−1.7; −0.61)	<0.001

The mean difference was calculated by comparing the baseline and follow-up values using a paired *t*-test.

**Table 3:** Linear mixed model analysis of factors influencing the amount of remodelling in Southwick angle.

Estimates of Effects	Est.	SE	t	*p*-Value	95% CI
Gender (male)	−1.2	1.1	−1.2	0.249	−3.5; 0.9
Age at surgery (years)	0.26	0.3	0.9	0.374	−0.32; 0.83
Follow-up duration (years)	0.36	0.3	1.4	0.158	−0.1; 0.9
Position screw on frog-leg lateral ^1^	−7.6	5.1	−1.5	0.140	−17.7; 2.5
**Southwick angle at baseline**	**0.8**	**0.0**	**18.9**	**<0.001**	**0.7; 0.9**

Bold values indicate statistical significance (*p* < 0.05). ^1^ 0 posterior; 100 anterior.

**Table 4:** Linear mixed model analysis of factors influencing the amount of remodelling of the Alpha angle.

Estimates of Effects	Est.	SE	t	*p*-Value	95% CI
Gender (male)	−0.2	1.7	−0.1	0.896	−3.6; 3.2
Age at surgery (years)	0.5	0.5	1.1	0.289	−0.4; 1.4
Follow-up duration (years)	−0.2	0.4	−0.5	0.617	−0.1; 0.6
Position screw on frog-leg lateral ^1^	6.8	8.0	0.8	0.397	−9.0; 22.7
**Alpha angle at baseline**	**0.8**	**0.1**	**14.4**	**<0.001**	**0.6; 0.9**

Bold values indicate statistical significance (*p* < 0.05). ^1^ 0 posterior; 100 anterior.

**Table 5:** Linear mixed model analysis of factors influencing the amount of remodelling in displacement from Klein’s line.

Estimates of Effects	Est.	SE	t	*p*-Value	95% CI
Gender (male)	−0.4	0.3	−1.2	0.227	−1.0; 0.26
Age at surgery (years)	0.0	0.1	0.1	0.930	−0.2; 0.2
Follow-up duration (years)	0.1	0.1	1.8	0.074	−0.0; 0.3
**Position screw on AP radiograph** **^1^**	**−4.2**	**1.9**	**−2.2**	**0.027**	**−8.0; −0.5**
**Displacement from Klein’s line at baseline**	**0.8**	**0.1**	**14.5**	**<0.001**	**0.7; 0.9**

Bold values indicate statistical significance (*p* < 0.05). ^1^ 0 medial; 100 lateral.

## Data Availability

All data generated or analysed during this study are available from the corresponding author upon reasonable request. The datasets are not publicly available due to privacy restrictions and ethical approval restrictions.

## Abbreviations

The following abbreviations are used in this manuscript:

FAI	Femoroacetabular impingement
SCFE	Slipped Capital Femoral Epiphysis
